# Serological Investigation and Molecular Detection of Newcastle Disease Virus From Apparently Healthy Village Chicken in East Gojjam Zone, Ethiopia

**DOI:** 10.1155/vmi/9933937

**Published:** 2026-07-22

**Authors:** Dessie Abera, Bezina Arega Emeru, Mezgebu Getnet, Beksisa Urge

**Affiliations:** ^1^ Animal Health Research Program, Ethiopian Institute of Agricultural Research, Debre Markos, Ethiopia, eiar.gov; ^2^ Animal Biotechnology Research Program, National Agricultural Biotechnology Research Center, Holeta, Ethiopia; ^3^ Animal Feed and Nutrition Research Program, Ethiopian Institute of Agricultural Research, Debre Markos, Ethiopia, eiar.gov; ^4^ National Animal Health Research Program, Holleta Agricultural Research Center, Holeta, Ethiopia

**Keywords:** hemagglutination inhibition test, Newcastle disease, RT-PCR, village chicken

## Abstract

Newcastle disease (ND) is a notifiable and highly contagious disease causing a significant economic loss in village chicken production. So, a cross‐sectional study was conducted in the East Gojjam zone of Ethiopia to determine the status of ND among unvaccinated village chickens. The study sites were selected randomly. However, the chicken owners included were households that had at least four chickens with no prior history of ND vaccination. A total of 314 serum and 76 pooled tracheal and cloacal swab samples were collected from apparently healthy birds. The hemagglutination inhibition (HI) test and reverse‐transcription polymerase chain reaction (RT‐PCR) were conducted for serological and virological tests, respectively. The data were analyzed using the SPSS version 20 software package. Descriptive statistics and generalized linear mixed models were used for data analysis. The overall seroprevalence rate at the individual chicken level was found to be 93% (292/314 chickens). At the household level, ND was identified in all sampled households, with a minimum of two seropositive chickens found in each household. However, these chickens tested negative for the ND virus by RT‐PCR. The seroprevalence rates of the NDV at each study site were 95.0%, 96.0%, and 86.9% for Gozamin, Enemay, and Debre Elias woredas, respectively, with a statistically significant difference (*p* < 0.05) observed among the study sites through descriptive analysis. Chickens from households where ND had occurred showed a significant association with seropositivity (*p* = 0.023, COR = 2.72). Contact with other flocks was the primary risk factor identified, with chickens that had such contact showing significantly higher odds of ND seropositivity compared to those that did not (*p* < 0.001, AOR = 5.18). Furthermore, households with access to veterinary service demonstrated significantly higher odds of NDV seropositivity (*p* = 0.006, AOR = 5.07) in comparison to those without such access. This implies that professional services in the area are utilized reactively in response to disease occurrence rather than as a proactive preventive measure. The findings revealed that the ND virus antibodies in village chicken were very high, indicating that ND is the major disease in the area that can threaten village chicken production. Therefore, programmed immunization is vital in order to prevent the occurrence of the disease. Awareness creation among smallholder farmers on the prevention and control measures for ND is recommended. Additionally, further research focusing on the virus strain identification is required.

## 1. Introduction

Poultry is the largest group of livestock species worldwide [1] that can contribute about 30% of all animal protein consumed in the world [2]. Village chickens are an integral part of smallholder farming. They are essential for the livelihood of the majority of households in developing countries [3]. It can also encourage HIV/AIDS mitigation and wildlife conservation programs. They are still important to millions after 8000 years of domestication [4]. However, poultry disease, particularly Newcastle disease, is a serious contagious poultry disease that causes significant economic losses in most poultry‐producing countries, including Ethiopia. The disease is widely spread and is the most challenging avian disease in the country [5]. ND is one of the major infectious diseases of village chickens in Ethiopia, threatening their survival and production potential [6]. The village chickens in Ethiopia are endemically infected with virulent NDV that poses a serious challenge to emerging small‐ and medium‐scale poultry productions [7]. It is notifiable to the World Organization for Animal Health (WOAH) and affects both domestic and wild bird species [8]. ND is caused by Newcastle disease virus (NDV). It became a serious threat to the poultry sector due to the emergence of antigenic variants of the virus [5]. ND alone can kill out the whole village flocks, and addressing this issue can improve the contribution of village chicken for the livelihood of individuals in the rural areas [9].

More than 90% of chickens in the country are raised in village production [10], and there is a high report of ND occurrence in rural areas where the ND vaccine is not delivered. Village chicken serves as a source for different strains due to the fact that village poultry management favors the existence and spread of diverse NDV strains by allowing free interaction of different poultry species and wild birds. No biosecurity measures were taken, and the frequent introduction of birds from markets aggravates the situation [8]. Knowing the status of ND in the area is critical to improve chicken production in low‐input rural settings, where it is a leading cause of chicken mortality. It is a prerequisite to designing effective control and prevention strategies. In addition, understanding the status of NDV in village chickens through serological and molecular investigations is scarce in Ethiopia [7]. And, there is no information about the status of ND in village chicken production in the East Gojjam zone, where there are many complaints of the disease. Therefore, this study was conducted to determine the seroprevalence of NDV through serological and molecular methods in unvaccinated village chickens.

## 2. Materials and Methods

### 2.1. Study Area Description

The study was conducted in the East Gojjam zone of Amhara National Regional State (ANRS), Ethiopia. East Gojjam is structured with 16 Weredas and four urban administrations. It is a diverse and agriculturally significant zone with 86% percent of the population living in rural areas relying heavily on agriculture as the main source of livelihood.

The area is geographically varied, with elevations ranging from 800 to 4200 m above sea level (m.a.s.l.), offering a variety of landscapes that influence local climate and agriculture. The East Gojjam zone experiences a unimodal rainfall pattern with the average annual rainfall ranging from 900 to 1800 mm, providing adequate water for crop cultivation. The annual average temperature ranges from a low of 7.5°C to a high of 27°C, reflecting its diverse topography, which includes both cooler highland and warmer lowland areas. Livestock production is a key part of the East Gojjam zone economy, with a significant number of different livestock species that account for a significant proportion of the ANRS livestock resource [11]. A population of livestock in the zone is as follows: cattle 2,185,995, sheep 1,225,349, goats 315,053, equine 571,867, poultry 1,087,572, and beehives 126,520. Compared to the ANRS livestock population, this is 12.6%, 11.9%, 4.6%, 12.4%, 6.7%, and 9.5% of the livestock resources available, respectively [10].

### 2.2. Study Design and Sampling Methods

A cross‐sectional study was conducted from May to June 2024 to investigate the status of ND virus in unvaccinated village chickens through seroprevalence and molecular detection methods. The chickens were free scavengers. The study woredas and kebelles were selected randomly. Yegagena and Asemabo from Gozamin, Yeqega and Sekela from Enemay, and Yeguarat and Tijagotir kebeles from Debre Elias woredas were included. A total of 86 households that had a minimum flock size of four chickens with no prior history of ND vaccination were selected randomly. Then, from each household, four chickens over 8 weeks old (> 2 months old) were sampled. The sample size was calculated based on the formula described by Thrusfield [12]. A country‐level meta‐analysis study of ND prevalence indicated that the overall prevalence was 22% [13], and by taking this as an expected prevalence, a minimum of 297 chickens were required, and a total of 314 chickens were sampled. The flock size was categorized into small (≤ 7 chickens), medium (8–10 chickens), and large flocks (> 10 chickens) for each household using the 33^rd^ and 66^th^ percentiles as (7 and 10 chickens, respectively) as a cut‐off value.

### 2.3. Sample Collection

Blood samples were collected from apparently healthy birds. About 1.5 mL of blood was collected from the brachial vein in a 3‐mL disposable syringe, using a new syringe and needle for each chicken. Blood was allowed to clot in a slant position and kept at room temperature for serum collection. The collected sera were stored at the Debre Markos Agricultural Research Center Laboratory until transported to the National Veterinary Institute (NVI), where the serological test was conducted.

A total of 76 pooled tracheal and cloacal swabs were collected for molecular investigation. Cryovials containing 2 mL of freshly prepared viral transport media (VTM) were used for tracheal (pool of 4) and cloacal (pool of 4) swabs separately. VTM containing antimicrobials like streptomycin, penicillin, and mycostatin in phosphate buffer solution was used. The swabs collected were transported in the icebox to the Debre Markos Agricultural Research Center laboratory. It was stored at −20°C until transported for ND virus detection to the National Agricultural Biotechnology Research Center. A standardized questionnaire was prepared and administered to the chicken owners to assess their experience in chicken handling, biosecurity practices, and disease history for risk factor determination.

Anesthetic was not administered during sample collection. The procedure is quick, and it causes minimal discomfort. Small volumes of blood were collected from the wing vein after proper restraining by standard venipuncture techniques. Swab sampling was a noninvasive procedure. All sampling procedures were carried out according to the ARRIVE 2.0 guidelines, where applicable to the prevalence study. Relevant ARRIVE 2.0 recommendations related to ethical handling of chickens during sample collection, sample description, and reporting transparency were followed to ensure full transparency and ethical compliance.

### 2.4. Serology

It is indispensable to know the status of the disease in the area. And, serological tests like the virus neutralization test, enzyme‐linked immunosorbent assay (ELISA), and hemagglutination inhibition (HI) test can be used for the diagnosis of infection of unvaccinated flocks [14, 15]. Serological detection of antibodies against the NDV was made according to the procedure in the OIE terrestrial manual 2018. Antibodies in serum are very stable to moderate environmental conditions. Standard protocols call for serum to be kept cold, but freezing of the sample is not necessary unless several weeks elapse between collection and testing [15]. Accordingly, the collected sera were stored at the Debre Markos Agricultural Research Center and then transported to the NVI. The serological test was conducted in the Deberzit National Veterinary Institute, Ethiopia, using laboratory procedures of the HI test.

The HI test was done following the procedures recommended by OIE [16]. The sera were inactivated in a water bath for 30 min at 58°C. The test was carried out in U‐bottomed microtiter plates by preparing twofold serial dilutions of equal volumes (0.025 mL) of phosphate‐buffered saline (PBS) and test serum (0.025 mL). Four hemagglutinating units (HAUs) of virus or antigen were added to each well, and the plate was maintained at room temperature for at least half an hour. Subsequently, 0.025 mL of 1% (v/v) washed chicken red blood cells were added to each well. The RBCs were gently mixed and allowed to settle for approximately 40 min at room temperature. The HI titer was determined from the highest dilution of serum that resulted in complete inhibition of 4 HAU of antigen. Agglutination was determined by tilting the plates, and only those wells with RBCs that streamed at the same rate as the control wells were considered positive for inhibition [16]. Full agglutination indicates a negative result. The cutoff titer for the test was 1:16. Thus, sedimentation of RBCs at the serum dilution of ≥ 1:16 indicates a positive reaction, and it was considered seropositive.

### 2.5. RNA Extraction and Polymerase Chain Reaction

The molecular detection assays were performed at the National Agricultural Biotechnology Research Center, Holleta, Ethiopia. The sample suspension in VTM was homogenized and processed for molecular characterization of NDV. The procedures and all the molecular detection methods conducted were according to Emeru and his colleagues’ report [14]. Then, 1 mL of a sample suspension was dispensed into a 1.5‐mL Eppendorf tube and centrifuged at 12,000 rpm for 3 min. The supernatant fluid was harvested, and RNA was extracted from 200, 150, and 100 µL of the supernatant for one, two, and three pooled samples using the DaAn Gene RNA purification kit (Guangzhou, China) following the manufacturer’s instructions. The extracted RNA was stored at − 80°C until processed. Reverse‐transcription polymerase chain reaction (RT‐PCR) was performed using the AccuPower Dual‐Hot Start RT‐PCR Kit (Bioneer, Korea) by targeting the Fusion gene conserved region of NDV. It was prepared according to Emeru and his colleagues’ report [17]. The forward primer was 5′ TCGCAAAATTATGGAGAAGC‐3′ and the reverse was 5′‐AGCAAGGTCTTTTGTTGTGC‐3′ with an amplicon product size of 386 bp. The reaction mixture containing the primers, the extracted RNA template, and nuclease free water were vortexed to create a homogenous mixture. Then, it was placed in a PCR machine (Mastercycler PCR thermal cycler, Germany). The PCR machine was set for thermal conditions of 48°C for 30 min for reverse transcription and initial denaturation at 95°C for 5 min. Then, amplification of the target gene was achieved by 36 cycles of denaturation at 94°C for 20 s, annealing at 58°C for 30 s, and extension at 72°C for 30 s with a final extension of 72°C for 10 min. Gel electrophoresis was performed to visualize the amplicons, and 1% agarose gel stained with ethidium bromide was prepared using a gel documentation system for the presence of the expected amplicon product.

### 2.6. Data Management and Analysis

Data entry and management were conducted with a Microsoft Excel spreadsheet. Statistical Package for the Social Sciences (SPSS) version 20 was used for data analysis. Descriptive statistics were used to calculate frequencies and percentages. The generalized linear mixed model, with households as a random effect, was used for both univariable and multivariable logistic regression analyses to identify various risk factors (explanatory variables) and to determine their association with the dependent variable (seroprevalence of the ND virus). Household was included as a clustering factor to account for the correlation between chickens available in a single household. The multivariable model was developed using a manual backward elimination strategy; the risk factors with a *p*‐value (< 0.25) in the initial univariable analysis were entered into the model, and nonsignificant variables were removed stepwise. Moreover, Akaike Information Criterion (AIC) and variance inflation factors (VIFs) were used to evaluate model fit and check for multicollinearity of explanatory variables, respectively. The chi‐squared test was also used to analyze the association between different parameters. The confidence level was held at 95%, and a value of *p* < 0.05 was considered statistically significant. However, during univariable analysis, a *p*‐value of < 0.25 was used for initial variable selection to avoid excluding important variables from the multivariable analysis [18].

### 2.7. Generative AI Statement

No generative AI tools were used in the preparation of this manuscript.

## 3. Results

The seroprevalence of ND at the individual chicken level was determined to be 93% (292/314) based on the results of descriptive analysis (Table 1). At the household level, ND was detected in all sampled households, with at least two seropositive chickens identified in each household. The seroprevalence of ND virus in each study site was determined. It was 95.0%, 96.0%, and 86.9% for Gozamin, Enemay, and Debre Elias woredas, respectively, and there was a statistically significant difference (*p* < 0.05) among the study woredas and kebeles. The highest seroprevalence was detected from Enemay (96.0%) and the lowest from Debre Elias (86.9%) woreda. Similarly, the highest seroprevalence was observed in Tijagotir (100.0%) and the lowest in Yeguarat (76.9%) kebeles.

**TABLE 1:** Seroprevalence rate of ND virus antibodies in village chickens in the study areas.

Study sites		Total no of tested samples	Seroprevalence rate (≥ 1:16)		Chi‐square (*p*‐value)
			Positive (%)	Negative (%)	
Woreda	Gozamin	121	115 (95.0)	6 (5.0)	6.07 (0.048)
	Enemay	101	97 (96.0)	4 (4.0)	
	Debre Elias	92	80 (86.9)	12 (13.1)	

Kebelle	Yegagena	54	53 (98.1)	1 (1.9)	20.7 (0.001)
	Asemabo	67	62 (92.5)	5 (7.5)	
	Yeqega	72	69 (95.8)	3 (4.2)	
	Sekela	29	28 (96.6)	1 (3.4)	
	Yeguarat	52	40 (76.9)	12 (23.1)	
	Tijagotir	40	40 (100.0)	0 (0.0)	

Total		314	292 (93.0)	22 (7.0)	

A univariable analysis was conducted by treating households as a random effect to identify potential risk factors for inclusion in the multivariable model. Risk factors with a *p*‐value of < 0.25 were considered for further analysis. Chickens from Enemay woreda showed a slight correlation with NDV seropositivity, indicating an increased risk. And, based on the age groups, the odds ratio of 0.51 in chick suggested that chicks were 49% lower odds of testing positive compared to adult groups, although these associations were not statistically significant. Chicken that had contact with other flocks were significantly associated with ND seropositivity (*p* < 0.001); they had 5.06 times the odds of being seropositive than the chicken that did not have contact with other flocks, and it is presented in Table 2.

**TABLE 2:** Univariable mixed‐effect logistic regression analysis of risk factors associated with ND seroprevalence.

Variable	Category	Number of tested chickens	Seroprevalence (%)	Crude OR (95% CI)	*p*‐value
Woreda^a^	Gozamin	121	95.0	2.37 (0.90–6.21)	0.081
	Enemay	101	96.0	2.71 (0.93–7.87)	0.067
	Debre Elias	92	86.9	Ref	—

Kebelles^a^	Yegagena	54	98.1	0.73 (0.07–7.39)	0.791
	Asemabo	67	92.5	0.34 (0.04–2.67)	0.306
	Yeqega	72	95.8	0.52 (0.06–4.31)	0.543
	Sekela	29	96.6	0.57 (0.05–6.22)	0.664
	Yeguarat	52	76.9	0.10 (0.01–0.67)	0.021
	Tijagotir	40	100.0	Ref	—

Sex	Male	54	94.4	0.81 (0.25–2.62)	0.723
	Female	260	92.7	Ref	—

Age^a^	Chick	48	87.5	0.51 (0.19–1.35)	0.175
	Adult	266	93.9	Ref	—

Flock size	≤ 7	135	94.1	0.65 (0.25–1.65)	0.361
	8–10	77	94.8	0.59 (0.19–1.83)	0.360
	≥ 10	102	90.2	Ref	—

Contact with other flocks^a^	Yes	209	98.1	5.06 (2.68–9.54)	0.000
	No	105	82.9	Ref	—

ND experience^a^	Yes	181	96.7	2.72 (1.15–6.46)	0.023
	No	133	87.9	Ref	—

Water source	Closed: tap/wells water	230	93.0	0.98 (0.38–2.53)	0.964
	Open: pond/river	84	92.9	Ref	—

Access to vet services^a^	Yes	284	94.4	3.60 (1.31–9.91)	0.013
	No	30	80.0	Ref	—

Dead bird disposal^a^	Thrown nearby	53	88.7	0.41 (0.13–1.28)	0.123
	Buried	71	88.7	0.41 (0.14–1.18)	0.098
	Thrown into a gorge or river	50	94.0	0.72 (0.19–2.75)	0.634
	Thrown far away	140	96.4	Ref	—

Abbreviations: CI = confidence interval, OR = odds ratio.

^a^Variable significance with *p* < 0.25 and selected for the multivariable mixed logistic regression model.

The size of the flock and the sex of the chickens did not show a statistically significant association with seropositivity. However, chickens from households where ND had occurred showed a significant association with seropositivity, being 2.72 times more likely to be positive than those from households without any occurrence of the disease. Chickens from a farm where ND occurred had a relatively higher seroprevalence (96.7%) than those that came from farms that had no ND occurrence (87.9%). Regarding the disposal of dead chickens, throwing them far away outside the compound poses the highest risk, whereas burying them (*p* = 0.098, COR = 0.41) was the most effective practice, reducing the odds of seropositivity by 59% compared to throwing them out of the compound.

A multicollinearity test was conducted prior to multivariable mixed‐effect logistic regression analysis. A significant multicollinearity was noted between the study woreda (VIF = 11.6) and kebelle (VIF = 11.8); separate models were evaluated to determine the most effective geographic predictor. The model that included woreda demonstrated a better fit (AIC: 1673.60 vs.1682.09) and higher classification accuracy (95.9% vs. 94.3%). Therefore, woreda was selected for the final multivariable analysis. Chickens from Enemay had around three times higher odds of being seropositive than chickens from Debre Elias, though not statistically significant (*p* = 0.090, AOR = 2.96). Higher seroprevalence was recorded in adults (94.0%) than in young (87.5%) (*p* = 0.075, AOR = 0.34) age groups. And, young chickens had 66% lower odds of being seropositive compared to adult age groups (Table 3).

**TABLE 3:** Multivariable mixed‐effect logistic regression analysis of risk factors associated with the seroprevalence of Newcastle disease.

Variable	Category	Number of chickens tested	Seroprevalence (%)	Adjusted OR (95% CI)	*p*‐value
Woreda	Gozamin	121	95.0	1.37 (0.45–4.15)	0.579
	Enemay	101	96.0	2.96 (0.84–10.37)	0.090
	Debre Elias	92	86.9	Ref	—

Age	Chick	48	87.5	0.34 (0.11–1.11)	0.075
	Adult	266	93.98	Ref	—

Contact with other flocks	Yes	209	98.08	5.18 (2.27–11.80)	0.000
	No	105	82.86	Ref	—

ND experience	Yes	181	96.7	2.05 (0.79–5.33)	0.140
	No	133	87.97	Ref	—

Access to vet services	Yes	284	94.4	5.07 (1.59–16.15)	0.006
	No	30	80.0	Ref	—

Dead bird disposal	Thrown nearby	53	88.7	0.70 (0.22–2.20)	0.540
	Buried	71	88.7	0.55 (0.18–1.66)	0.285
	Thrown into a gorge or river	50	94	0.99 (0.25–3.89)	0.984
	Thrown far away	140	96.4	Ref	—

Contact with other flocks has a significant association with the seroprevalence of ND (*p* < 0.001, AOR = 5.18), and chickens that had contact with other flocks have 5.18 higher odds of being seropositive than chickens that did not have contact with other flocks. The ND experience in their chicken farms was significantly associated (*p* = 0.023, COR = 2.72) with seropositivity in the univariable analysis. However, in the multivariable analysis, it becomes nonsignificant (*p* = 0.140). Households who had access to veterinary service had significantly higher odds of NDV seropositivity (*p* = 0.006, AOR = 5.07) compared to those without such access. According to the dead chicken disposal methods practiced in the area, burying them (*p* = 0.285, COR = 0.55) results in a 45% lower odds of being seropositive compared to those who throw them far away.

**FIGURE 1:**
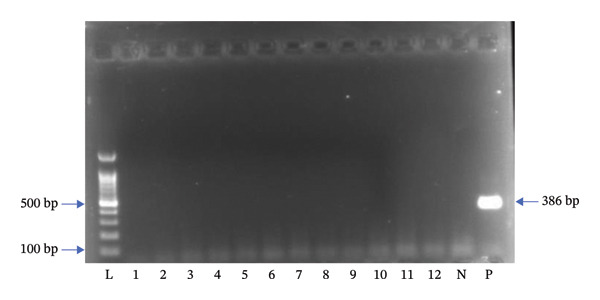
Gel image. Legend: ‐ lane L: ladder (marker 100 bp); lanes 1–12: samples; lane N: negative control; and lane P: positive control.

A total of 76 pooled swab samples were examined by RT‐PCR by targeting the Fusion (F) gene conserved region of the ND virus. However, they were negative for the virus (Figure 1).

## 4. Discussion

The high seroprevalence recorded in the current study shows that ND is the most important constraint of village chicken production in the area. From the total of 314 serum samples tested by using the HI test, 292 (93.0%) samples were seropositive for the ND virus antibodies. This high level of seropositivity indicates the endemic status of the disease in the area. This finding aligns with the report of Chaka and his colleagues from the Eastern Showa Zone, who stated that the lack of preventive and control measures to combat the disease in village chicken production has contributed to the spread and persistence of ND infections [7]. The results further support the hypothesis that low virulent NDV strains circulate continuously within scavenging village chickens, leading to repeated natural exposure and long‐term persistence of antibodies [19, 20]. Given that maternally derived ND antibodies protect chickens against the disease up to 2 weeks of age only [21, 22], the high level of ND antibody titers observed in unvaccinated, older chickens in this study suggests the presence of circulating field strains of the ND virus in the study area, indicating a natural infection. Furthermore, the sampling period (May–June) coincides with the transition to the rainy season in Ethiopia, which is a period characterized by nutritional scarcity and environmental stress, which may exacerbate chickens’ susceptibility to ND infection. These findings are consistent with Haile and Fentie [23], who reported a relatively higher prevalence of ND during the pre‐rainy (April–May) season of the year in Northwest Ethiopia.

The finding of this study was comparable with the report of Birhan et al. [24]; 92.4% seropositivity was detected in the Dembiya district, North Gondar, Ethiopia. However, it was higher than the report of Tadesse and his colleagues from Central Ethiopia; the overall seropositivity rate was 32.22% [6]. This might be related to the difference in awareness and practices of poultry keepers to control and prevent the disease. Poor biosecurity measures and the absence of vaccination in the area may contribute, and Central Ethiopia is better compared to rural areas of the country for accessing various training programs and other disease control and prevention packages. In addition, Haile and Fentie from Northwest Ethiopia [23], Emeru, and his colleagues from Southwest Ethiopia [25], Mamo and Yimer from Bunobedelle zone, Western Ethiopia [26], and Biswas and his colleagues from Bangladesh [27] have reported that the overall prevalence of NDV antibodies was 38.9%, 68.8%, 30.0%, and 88%, respectively. The seroprevalence of ND antibodies from backyard chickens was also reported from the Rift Valley areas, 11.6% [28], and the Gofa Zone of Southwest Ethiopia, 68.8% [29]. The finding of this study was lower than the report from Northwestern Ecuador; 97% of backyard chickens had antibodies against the ND virus [30]. The difference in the seroprevalence of ND between the findings from different geographic areas might be related to the difference in the breed of chicken, age, management practices, climatic conditions, season, and geographic location [7, 19].

In the current study, based on univariable descriptive analysis, study sites were statistically associated (*p* < 0.05) with NDV seropositivity. However, they lost significance in the mixed‐effect logistic regression analysis after considering household as a clustering factor. This suggests that the geographic variation observed was probably influenced by particular high‐risk management practices within individual households, rather than by broader environmental or administrative differences among study sites. Among the risk factors assessed, the sex of chickens was another risk factor considered, and numerically higher seroprevalence was detected in male (94.4%) than in female (92.7%) chickens. But there was no statistically significant association (*p* > 0.05) with seroprevalence of the ND virus. This finding is comparable with the report of Getachew and his colleagues from Southern Tigray; the disease prevalence has no statistically significant variation between sexes (*p* = 0.415) [31]. Similarly, a report from the Oromia region indicated that the difference was not statistically significant (*p* = 0.337) based on the sex of chickens [32]. However, in another study conducted in Southwestern Ethiopia, the seroprevalence was higher (*p* = 0.007) in female (20.3%) than in male (8.3%) chickens [33]. Similarly, another scholar from Southwest Ethiopia reported that significantly higher seroprevalence (*p* < 0.001) was reported in female (76%) than in male (33%) chickens [29]. The sex and the flock size of the chickens did not show a statistically significant association with ND seropositivity in this study. This indicates that the management practices, such as contact with other chickens, are more influential than the number of chickens or their sex within households.

Higher seroprevalence was recorded in adults (94.0%) than in young (87.5%) (*p* = 0.075) age groups in this study. It is comparable with the report from Southwest Ethiopia, who noted that adult chickens are more likely to get infection [25]. The seropositivity difference between young and adult chickens became higher in the multivariable mixed‐effect logistic regression analysis (COR = 0.51, AOR = 0.34). The lower odds of being seropositive of young chickens increased (AOR = 0.34) compared to the univariable estimate (COR = 0.51). This might be due to more frequent exposure of adult chickens to the field viral strains. They have a relatively higher chance of contact with wild birds, scavenge from contaminated areas, or interact with other infected flocks [33]. In addition, various household‐level risk factors may have been confounding the association between age and ND seropositivity, and in the multivariable analysis, young chickens were less likely to be seropositive than adults. It is likely attributable to their shorter duration of environmental exposure to the ND virus. However, there is a report from Southwest Ethiopia that higher seroprevalence was reported in the young (58.0%) than the adult (42.0%) age groups [29].

Regarding contact with other flocks, chickens that had contact with other flocks were higher (98.1%) in seropositivity than those chickens that did not have contact (81.9%). It has statistically significant association with the seroprevalence of ND (*p* < 0.001, AOR = 5.18). Contact between flocks was identified as a primary risk factor in this study, and it highlights the role of free scavenging in disease transmission in village chicken production. Similar findings have been reported by many scholars [6, 21, 34, 35], who pointed out that the open scavenging nature of village production facilitates the rapid spread of the virus. This finding was also comparable with the report from the Wolaita Zone of Southern Ethiopia; contact with neighboring chickens was the potential risk factor that was statistically associated (*p* < 0.05) with the ND seropositivity [21]. In addition, scholars from Sudan indicated that NDV seropositivity in village chickens was significantly associated with contact with neighboring chickens [35]. The significance of management factors, along with the loss of significance of study sites in the multivariable mixed‐effect logistic regression, emphasizes that household‐level biosecurity is a key determinant of NDV exposure in the study area.

Regarding ND occurrence in their farms, the chickens from households where ND had occurred were significantly associated (*p* = 0.023) with seropositivity in the univariable analysis. However, it lost its significance in the final multivariable analysis (*p* = 0.140). This indicates that the risk previously attributed to the history of ND in the flock was influenced by other variables. Households that had access to veterinary services in chicken health management had significantly higher odds of NDV seropositivity (*p* = 0.006, AOR = 5.07) compared to those without such access. The higher seroprevalence observed among households utilizing veterinary services might be associated with those within the study area, who could be reactive to disease outbreaks and therefore seek veterinary services during or after disease occurrence in their chicken farms. This finding aligns with the report of Asfaw and his colleagues [36], who reported that utilization of veterinary services by smallholder poultry farmers was associated with the history of disease occurrence in their chicken flocks. However, other scholars from different parts of Ethiopia reported that the difference in veterinary service has no statistically significant association (*p* = 0.35) with seropositivity from Southwest Ethiopia [29]. And, Emeru and his colleagues from the Central and Southwestern areas of Ethiopia indicated that access to veterinary service significantly affects seropositivity, and it was highly likely in reducing susceptibility to ND (*p* = 0.000) [17]. The high seroprevalence observed (93%) in the absence of a proactive vaccination program in this finding indicates a widespread natural circulation of the ND virus within a study area. Furthermore, the significant association of seropositivity with veterinary access suggests that these services are utilized reactively.

In the present study, the source of water provided to chickens was not statistically associated with seropositivity. However, Wodajo and his colleagues from Southern Ethiopia reported that the source of water for chicken was statistically associated (*p* < 0.05) with ND seropositivity [21]. And, a report from Eastern Shewa Zone, Ethiopia, indicated that the source of the water was significantly associated (*p* < 0.05) with seropositivity [34]. The analysis of dead chicken disposal methods revealed that, particularly, burying showed a protective trend in the univariable analysis (*p* = 0.098, COR = 0.41) compared to other disposal practices. However, it lost its significance (*p* = 0.285, COR = 0.55) in the multivariable analysis. This might be due to the fact that the benefit of proper disposal is confounded by other variables. Additionally, in an environment where seroprevalence is high (93%), the environmental viral load and communal transmission routes might outweigh individual household actions, such as burying carcasses.

Regarding molecular detection, all the tracheal and cloacal swab samples tested using RT‐PCR by targeting the Fusion (F) gene conserved regions of ND virus in the present study were negative. However, many scholars have reported the availability of different genotypes of the virus in different parts of the country [7, 17, 19, 20, 32]. This negative result for the F gene of the ND virus in this study might be due to the fact that apparently healthy chickens were sampled, and they may not have been shedding the virus, and/or no active viral replication was occurring at the time of sampling. Furthermore, a single cross‐sectional sampling of apparently healthy chickens might have failed to detect intermittent shedding of the ND virus. This is due to the fact that chickens may exhibit discontinuous or intermittent NDV shedding, which can be influenced by the infection stage and the immune status of the host [37]. The chickens may also have recovered from previous NDV infection, and HI detects antibodies that can persist in the chicken after exposure. This is in agreement with Biswas and his colleagues, who described that the HI titer specific to NDV is a sign of previous infection in nonvaccinated chickens [27].

## 5. Limitation of the Study

This study employed a cross‐sectional study design, and we are unable to determine the exact timing of ND infection to establish a definitive causal relationship between the identified risk factors and the timing of viral introduction into the flock.

## 6. Conclusion

The study indicated that village chickens have been significantly exposed to the ND virus, with an overall seroprevalence of 93.0% among unvaccinated chickens. This underscores the importance of the disease in village chicken production of the study area. The analysis conducted using multivariable mixed‐effect logistic regression demonstrated that contact with other flocks was identified as a potential risk factor for the ND seropositivity. This highlights the significance of the free scavenging behavior of village chickens in the transmission of the disease. Thus, transmission of ND may be primarily driven by household‐level management practices, such as contact with other flocks that could act as a bridge for the ND virus spread in the area. Additionally, veterinary access was also statistically associated with the ND seropositivity, indicating that professional services are being utilized reactively in response to disease occurrence rather than proactively for prevention in the area. Therefore, it is essential to create awareness among smallholder farmers about the proper handling of chickens to prevent and control infectious diseases. Village chickens need to be vaccinated against ND, and veterinarian interventions should be more proactive than reactive for the prevention of disease occurrence. Moreover, further studies on ND virus strain identification are recommended to identify the circulating virus genotypes.

## Author Contributions

Dessie Abera conceptualized the study, methodology, investigation, formal analysis, and writing the original draft. Bezina Arega Emeru conducted investigation, data curation, review, and editing. Mezgebu Getnet performed methodology, investigation, review, and editing. Beksisa Urge conducted supervision, methodology, analysis, review, and editing.

## Funding

This research study did not get any specific funding. But the Ethiopian Institute of Agricultural Research provided all the required inputs.

## Disclosure

All authors read and approved the final version of this manuscript.

## Ethics Statement

This study was approved by the Animal Research Ethics Committee of Debre Markos Agricultural Research Center. The ethical clearance was obtained from the committee with a certificate reference number of EIAR/ERC/006/2024. The study was conducted according to the ethical standards and principles of the research practice of the Ethiopian Institute of Agricultural Research. Chickens were handled with kindness and proper care by minimizing discomfort, distress, and pain during sample collection. It was carried out according to the ARRIVE 2.0 guidelines where applicable.

## Conflicts of Interest

The authors declare no conflicts of interest.

## Data Availability

The data supporting this study are available from the corresponding author and can be shared if required.
